# Phytobiotic-enriched multiphase feeding improves growth and reproductive performance in Manchurian quails: Evidence from controlled farm trials in Kazakhstan

**DOI:** 10.14202/vetworld.2025.3120-3134

**Published:** 2025-10-26

**Authors:** Dinara Zhanabayeva, Botagoz Aitkozhina, Gulmira Abulgazimova, Dilora Senkebayeva, Bakytkanym Kadraliyeva, Saidulla Ruzmatov, Assylbek Zhanabayev

**Affiliations:** 1 Department of Veterinary Sanitation, NCJSC “S. Seifullin Kazakh Agro Technical Research University,” Astana City, Zhenis Avenue 62, Republic of Kazakhstan; 2 Department of Veterinary Medicine, NCJSC “S. Seifullin Kazakh Agro Technical Research University”, Astana City, Zhenis Avenue 62, Republic of Kazakhstan; 3 Department of Technology of Production and Processing of Animal Products, NCJSC “S. Seifullin Kazakh Agro Technical Research University”, Astana City, Zhenis Avenue 62, Republic of Kazakhstan; 4 Institute of Veterinary Medicine and Agricultural Technology, Zhangir Khan Agrarian Technical University, Uralsk City, Zhangir Khan 51, Republic of Kazakhstan

**Keywords:** egg production, feed additives, growth performance, phytobiotics, quail farming, sustainable poultry nutrition

## Abstract

**Background and Aim:**

Manchurian quails are valued in commercial poultry farming for their early meat maturity, rapid generational turnover, and high egg-laying potential. However, concerns over antibiotic use in feed have heightened interest in sustainable alternatives such as phytobiotics. Despite evidence supporting phytogenic feed additives, limited studies have examined their effects in Manchurian quails under multiphase feeding regimens. This study evaluated the effects of phytobiotic-enriched, extruded feeds (BioFeed-P) across three feeding phases (“Starter,” “Grower,” and “Layer”) on growth performance, egg productivity, and product quality in Manchurian quails.

**Materials and Methods:**

A total of 1600 1-day-old quails were randomly allocated into experimental group (EG) and control group (CG) at two commercial farms (JEBE; Zhailybayev Experimental Breeding Enterprise and ECO-KO; Ecological Cooperative Kazakhstan Organization)in Kazakhstan. EG received phytobiotic-enriched multiphase feeds, whereas CG was provided standard commercial diets. Growth rate, feed conversion ratio (FCR), survivability, and egg production were measured over a 90-day period. Product quality was assessed by analyzing egg morphology, chemical composition, and mineral profiles. Statistical analyses included the Student’s t-test, correlation analysis, and determination of effect size.

**Results:**

Quails in EG showed significantly higher body weight gain (186.3 g vs. 135.3 g; relative gain 415.5% vs. 297.5%; p = 0.003, Cohen’s d = 1.12) and improved FCR (2.05 vs. 2.45; p = 0.001). Egg-laying intensity was greater in EG at both ECO-KO (64.4% vs. 41.3%; p = 0.004) and JEBE (69.0% vs. 40.0%; p = 0.003). Egg morphology showed modest changes: Heavier albumen, reduced shell weight, and lighter yolk pigmentation. Chemical analyses revealed slight decreases in protein and fat fractions accompanied by minor increases in carbohydrate and ash content, while the mineral composition remained stable. Mortality was lower in EG (3.8% vs. 7.7%), with no adverse health effects.

**Conclusion:**

Phytobiotic-enriched multiphase feeds significantly enhance growth efficiency and reproductive output in Manchurian quails without compromising mineral egg quality. These findings support phytobiotics as sustainable alternatives to antibiotics in commercial quail farming. Future research should include long-term reproductive assessments, economic cost–benefit analyses, and molecular studies to elucidate the underlying mechanisms.

## INTRODUCTION

In recent years, poultry farming has emerged as one of the fastest-growing sectors of the animal husbandry industry. Poultry meat, due to its high protein content, is regarded as a diabetic-friendly product [1], whereas eggs provide considerable amounts of B-group vitamins and functional enzymes, including proteases, diastases, and dipeptidases [2]. Within this context, quail farming represents a relatively new yet highly promising branch of poultry production [3, 4]. However, the widespread use of artificial feed additives, antimicrobial agents, and antibiotics in poultry nutrition has raised concerns due to declining product quality, food safety risks, and the emergence of antimicrobial resistance [5, 6]. As an alternative, a study by El-Saadony *et al*. [7] on bioactive peptides, such as those derived from peas and red kidney beans, has demonstrated their potential to enhance the stability and safety of animal-derived foods, including buffalo meat during refrigeration.

According to 2024 statistics, Kazakhstan alone hosts over 100 quail farms, maintaining a population of more than 4 million birds. This expansion highlights the substantial economic potential of quail farming, where sustainable and cost-effective nutritional strategies, particularly phytobiotic supplementation, can improve profitability compared to traditional antibiotics or conventional feeds [8–10].

Phytobiotics, also known as phytogenics or plant-based preparations, have gained considerable attention as natural protein sources and alternatives to antibiotics in poultry diets [11]. Although relatively new in poultry nutrition [12], phytobiotics are increasingly investigated for their ability to sustainably enhance growth performance, bird health, and product quality. Derived from herbs, spices, plants, and their extracts (including essential oils), these supplements contain a diverse range of bioactive compounds with beneficial effects on productivity [13–16]. In quails, phytogenic additives have been shown to enhance egg quality, improve production efficiency, and increase feed utilization, thereby reinforcing their role as natural substitutes for antibiotics [17]. For instance, supplementation with *Yucca schidigera* extract improved reproductive performance, immune responses, antioxidant capacity, and overall health in Japanese quails exposed to dietary lead [18].

Evidence further indicates that phytobiotics enhance growth rates, feed conversion, and egg quality through antifungal, anti-inflammatory, and antioxidant mechanisms [19], while also reducing feed contamination and promoting gut health [20–24]. Other natural strategies, such as integrating probiotics or prebiotics with live vaccines, have also demonstrated synergistic benefits; for example, the co-administration of a Salmonella vaccine in broilers improved growth and reduced pathogen shedding [25]. Collectively, phytogenic additives strengthen poultry health and performance by leveraging antimicrobial and antioxidant pathways, thereby supporting sustainable production systems and ensuring consumer safety [26].

Despite growing evidence on the benefits of phytobiotic feed additives in poultry, current studies remain limited in scope and application. For instance, a 60-day trial in Texas quails demonstrated that phytobiotic-extruded feed improved growth performance, immune function, and feed conversion efficiency [27]. Similarly, supplementation of layer quail diets with phytogenic additives significantly enhanced egg production, eggshell quality, and gut health when compared with antibiotic-based regimens [28]. However, these studies primarily focused on single-phase feeding or short-term supplementation, without addressing the effectiveness of integrated multiphase regimens tailored to the physiological needs of different growth and reproductive stages. Furthermore, little is known about the specific responses of Manchurian quails, a breed less frequently studied compared to Japanese or Texas quails, under such feeding systems. This gap highlights the need for comprehensive research evaluating phase-specific phytobiotic-enriched feeding strategies that could optimize both growth and reproductive outcomes while maintaining product quality and safety.

To address this gap, the present study aimed to develop and evaluate phytobiotic-enriched extruded feeds designed for three distinct phases of quail rearing –”Starter,” “Grower,” and “Layer.” By applying these phase-specific diets under controlled farm conditions in Kazakhstan, the study sought to assess their impact on live weight gain, feed efficiency, survivability, and egg production. In addition, the research examined egg morphological characteristics, chemical composition, and mineral content to determine whether phytobiotic supplementation altered product quality. Through this approach, the study not only investigated the growth-promoting and reproductive benefits of phytobiotics in Manchurian quails but also generated practical recommendations for sustainable quail farming that minimize reliance on antibiotics and artificial additives.

In addition to the growing focus on phytobiotic supplementation, this study has also emphasized feed-based therapeutic formulations aimed at improving animal health and parasite control. For example, Patent No. KZ21292-A4 describes feed granules composed of fenbendazole, wheat, and barley for the treatment of helminthiasis in horses. Such patented formulations demonstrate the potential of combining nutritional and therapeutic strategies to enhance livestock performance, immunity, and overall resilience [29].

## MATERIALS AND METHODS

### Ethical approval

The experimental protocol was approved by the Ethics Committee of Non-Commercial Joint Stock Company (NCJSC) “S. Seifullin Kazakh Agro Technical Research University” (Protocol No. 2, December 14, 2021). The study adhered to Animal Research: Reporting of *In Vivo* Experiments (ARRIVE 2.0) guidelines, including randomization, blinding of outcome assessment, and humane endpoints. All procedures complied with national animal welfare regulations and followed international standards, including those of the World Organization for Animal Health.

### Study period and location

This controlled, field-based feeding trial was conducted between 2022 and 2024 at two commercial quail farms in the Republic of Kazakhstan: JEBE; Zhailybayev Experimental Breeding Enterprise and ECO-KO; Ecological Cooperative Kazakhstan Organization. Laboratory analyses of feed, eggs, and meat were performed at the Almaty Technological University laboratories.

### Study design

This study employed a controlled, randomized experimental design to evaluate the effects of phytobiotic-enriched extruded feeds (BioFeed-P) on the growth and egg productivity of Manchurian quails under practical farming conditions.

### Experimental birds and grouping

A total of 1600 1-day-old Manchurian quail chicks were allocated into two groups using computer-generated randomization (block size = 50 chicks) and stratified by farm:

- Experimental group (EG): 800 birds
- Control group (CG): 800 birds.

Each farm received an equal number of birds per group:

- JEBE Farm: EG (n = 400), CG (n = 400)
- ECO-KO Farm: EG (n = 400), CG (n = 400).

Within each farm, birds were subdivided into four replicates of 100 birds per replicate and housed in separate cages. Replicate cages served as the experimental unit. A computer-based random sequence was used for cage allocation. Although no formal sample size calculation was performed, the replicate design ensured sufficient statistical power.

### Feed formulations and feeding regimens

Three specialized phytobiotic-enriched feed formulations were designed for distinct growth phases:

- Starter feed: 0–14 days
- Grower feed: 14–45 days
- Layer feed: 36+ days.

Feeds were prepared in accordance with the nutrient requirements of ring-necked pheasants, Japanese quails, and Bobwhite quails [30]. All phytobiotic feeds were extruded and contained BioFeed-P at phase-specific concentrations (0.4%–0.5%).

- EG (Experimental Group): Received the Starter, Grower, and Layer feeds sequentially.: Received the Starter, Grower, and Layer feeds sequentially.
- CG (Control Group): Received standard commercial compound feeds used at each farm:
  - Broiler Meat Developer Vitamin (BMDV) 15% (TOO AgroFeed, Almaty, Kazakhstan) at JEBE Farm
  - AVA CHICK (Commercial feed from AVA Feed Mills, Kostanay, Kazakhstan) at ECO-KO Farm

Feeds were developed in collaboration with Almaty Technological University, whereas control diets were sourced commercially. Feed and water were provided ad libitum. Feed intake was recorded per replicate cage, and the feed conversion ratio (FCR) was calculated as feed intake (g) divided by body weight gain (g).

### Phytobiotic composition

The phytobiotic-enriched feeds included plant species rich in bioactive compounds:

- Thyme (*Thymus vulgaris*), garlic (*Allium sativum*), and oregano (*Origanum vulgare*), supplying thymol, allicin, and flavonoids.
- Inclusion rates: Thyme extract (0.5%), garlic powder (1%), and oregano extract (0.3%) at phase-specific concentrations.
- BioFeed-P composition: Standardized proprietary blend containing thymol (0.2%), allicin (0.15%), and total flavonoids (0.3%) measured by HPLC.

These compounds are recognized for their antimicrobial, antioxidant, and anti-inflammatory effects, enhancing overall poultry health and productivity.

### Housing and environmental conditions

Birds were housed in environmentally controlled cages under standardized conditions:

- Light cycle: 16 h light/8 h dark
- Temperature: 21°C–24°C
- Relative humidity: 55%–65%
- Ventilation: Automated airflow system.

Environmental parameters (temperature, humidity, and light intensity) were continuously monitored using thermometers, hygrometers, and lux meters. Conditions were maintained consistently to minimize environmental variation affecting growth and productivity.

### Chemical and nutritional analyses

#### Feed analysis

- Proximate composition was determined using a FOSS 2500 infrared analyzer (FOSS Analytical, Denmark).
- Amino acid profile analyzed using capillary electrophoresis (Kapel-105 system, Lumex Instruments, Russia) according to Gosudarstvennyy Standard: State Standard of the Russian Federation (GOST) 32299–2013 and International Organization for Standardization (ISO) standards [31, 32].

#### Quail product composition

- Muscle tissue composition analyzed using Association of Official Analytical Chemists (AOAC) protocols and instruments such as Atomic Absorption Spectrophotometer and Inductively Coupled Plasma–Optical Emission Spectrometry (Agilent Technologies, USA).
- Egg ash content determined by GOST 15113.8–77.
- Mineral composition of meat and eggs evaluated using GOST 32343–2013, GOST 9794–2015, and GOST 31707–2012.

### Productivity Assessment

#### Growth performance

Weekly measurements of body weight and feed intake were collected. Growth rate, FCR, and survivability were calculated using standard poultry performance formulas.

#### Egg production

Egg-laying performance was assessed by recording:

- Age at onset of laying
- Daily egg count per bird
- Average egg mass.

Egg productivity was calculated as a composite index of laying rate and egg mass. If the laying rate was identical, a greater average egg mass was considered superior.

### Statistical analysis

Data were analyzed using the Statistical Package for the Social Sciences v.25.0 (IBM Corp., NY, USA). The Shapiro–Wilk test was applied for normality. Differences between EG and CG were tested using Student’s t-test (two-tailed), with Levene’s test applied to confirm variance equality. Analyses were conducted per replicate cage to account for clustering. Significance was set at p ≤ 0.05. Results are presented as mean ± standard deviation, with 95% confidence intervals (CI) and Cohen’s d values.

## RESULTS

### Mortality and health records

**Figure 1:**
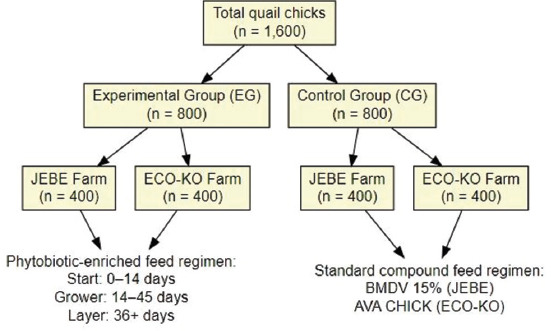
Experimental design and allocation of 1600 Manchurian quail chicks into experimental and control groups across two farms (JEBE and ECO-KO), with corresponding feeding regimens and replicates per cage.

The overall survival of Manchurian quails was high in both groups. The mortality was slightly lower in the EG at 3.8% (1/26) compared to 7.7% (2/26) in the CG (Figure 1). No significant morbidity or adverse health events were recorded, and all birds remained clinically healthy throughout the study period.

### Nutrient composition of feeds

Table 1 illustrates differences in nutrient composition among commercial and phytobiotic-enriched feeds. Feed formulation details and grouping design are presented in Figure 1. Commercial feed no. 2 had higher crude protein (25.4%) and fat (6.9%) compared to commercial feed no. 1 (23.0% protein, 6.1% fat). Among phytobiotic feeds, the Starter feed contained 27.6% crude protein, whereas the Grower feed provided the highest protein content (26.9%) and metabolic energy (3371 kcal), exceeding that of the Layer feed (25.6% protein). These values suggest that the Grower feed was nutritionally optimized for growth enhancement.

**Table 1:** Nutrient content in commercial and developed quail feeds.

Feed	Humidity	Crude protein	Raw fat	Crude fiber	Starch	Ash	Metabolic energy (birds), kcal
Commercial Feed No. 1	10.0	23.0	6.1	4.07	32.17	6.04	3291
Commercial Feed No. 2	10.4	25.4	6.9	3.45	33.13	4.93	3331
Starter	9.2	27.6	7.1	3.72	26.01	6.55	3347
Grower	8.0	26.9	7.6	2.78	31.11	6.79	3371
Layer	8.9	25.6	7.1	2.8	33.21	5.83	3348

Values expressed as %; ME = Metabolic energy, CG = Control group, EG = Experimental group.

### Growth performance

**Figure 2:**
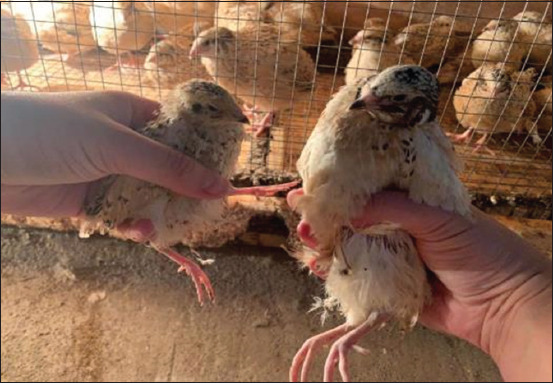
Representative quails aged 35 days from the control (left, fed commercial feed) and experimental (right, fed phytobiotic grower feed) groups.

**Figure 3:**
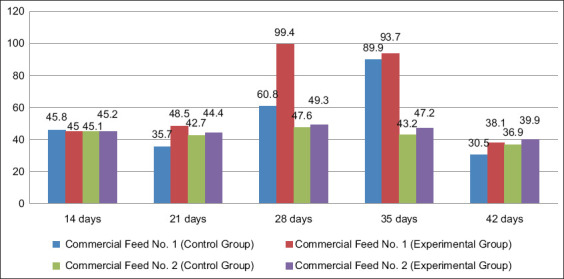
Absolute gain (g) in Manchurian quails at different ages (14, 21, 28, 35, and 42 days) under various feed formulations (Commercial feed 1, Commercial feed 2, and phytobiotic Grower feed). The error bars represent the standard error of the mean.

Manchurian quails fed photobiotic-enriched feeds exhibited superior growth compared with those receiving commercial diets. Representative growth differences between control and experimental birds are shown in Figure 2, while growth trends across ages and feed types are illustrated in Figure 3. As shown in Table 2, absolute weight gain was significantly higher in the EG (186.3 g) than in the CG (135.3 g) (p = 0.003, 95% CI: 0.98–2.56, Cohen’s d = 1.12). Average daily gain peaked at 14.2 g in the EG at 28 days compared with 12.8 g in the CG at 35 days. Relative gain was also significantly higher in the EG (415.5%) versus the CG (297.5%) (p = 0.002, Cohen’s d = 1.05).

**Table 2:** The growth performance (mean ± SEM) of Manchurian quails fed with commercial feeds versus phytobiotic-enriched Grower feed.

Age (days)	Indicator	Commercial feed No. 1		Commercial feed No. 2	
	
		CG	EG (grower)	(CG)	EG (grower)
14	Live weight (g)	45.8 ± 0.5	45.0 ± 0.4	45.1 ± 0.9	45.2 ± 0.7
21	Live weight (g)	81.5 ± 1.0	93.5 ± 1.1*	86.5 ± 3.1	89.6 ± 1.2*
	Absolute gain (g)	35.7 ± 1.3	48.5 ± 1.1*	42.7 ± 2.7	44.4 ± 1.4*
	Average daily gain (g)	5.1 ± 0.2	6.9 ± 0.2*	6.1 ± 0.4	6.4 ± 0.2*
28	Live weight (g)	99.5 ± 4.7	147.9 ± 3.0*	135.3 ± 2.7	138.9 ± 2.7*
	Absolute increase (g)	60.8 ± 6.1	99.4 ± 3.0*	47.6 ± 3.4	49.3 ± 2.32*
	Average daily gain (g)	8.7 ± 0.9	14.2 ± 0.4*	6.8 ± 0.5	7.1 ± 0.3*
35	Live weight (g)	150.6 ± 4.6	193.1 ± 2.9*	178.5 ± 2.4	186.3 ± 2.6*
	Absolute gain (g)	89.9 ± 6.6	93.7 ± 4.3*	43.2 ± 3.2	47.2 ± 3.1*
	Average daily gain (g)	12.8 ± 0.9	13.4 ± 0.6*	6.2 ± 0.5	6.8 ± 0.5*
42	Live weight (g)	181.1 ± 6.3	231.3 ± 3.3*	215.4 ± 2.7	225.6 ± 4.4*
	Absolute gain (g)	30.5 ± 8.0	38.1 ± 4.2*	36.9 ± 3.2	39.9 ± 3.2*
	Average daily gain (g)	4.4 ± 1.1	5.4 ± 0.6*	4.4 ± 1.1	5.3 ± 0.5*
Total	Absolute gain (g)	135.3 ± 6.7	186.3 ± 3.3*	170.3 ± 2.7	180.2 ± 3.6*
	Average daily gain (g)	6.1 ± 0.2	6.7 ± 0.1*	6.1 ± 0.1	6.4 ± 0.1**
	Relative gain (%)	297.5 ± 15.6	415.5 ± 7.8*	382.6 ± 9.9	403.9 ± 10.6*

* indicates a significant difference compared with the corresponding control (p < 0.05). CG = Control Group, EG = Experimental Group, SEM = Standard error of the mean.

Feed efficiency was markedly improved, with EG showing a lower FCR (FCR: 2.05 ± 0.08) than CG (2.45 ± 0.12; p = 0.001).

### Between-farm reproducibility

Consistent patterns were observed across both farms. At JEBE, EG quails showed a 23.0% greater body weight gain compared to CG, whereas at ECO-KO the increase was 21.5%. These findings suggest reproducibility of phytobiotic effects under different farm conditions, though with moderate variability.

### Egg production and laying performance

Egg productivity results (Table 3) demonstrated significant improvements in EG compared to CG. At ECO-KO, the EG produced 1488 eggs, whereas CG laid only 953 eggs (p = 0.002, Cohen’s d = 1.12). At JEBE, EG laid 1533 eggs compared with 867 in CG (p = 0.001, Cohen’s d = 1.25).

**Table 3:** The egg productivity (number of eggs) and laying intensity (%) of Manchurian quails at the ECO-KO and JEBE farms under different feeding regimes. Results are expressed as mean values over 90 days, with indicated significance levels.

Indicator	Measure	ECO-KO		JEBE	
	
		CG (C.F. 2)	EG (starter + grower + layer)	CG (C.F. 2)	EG (starter + Grower + layer)
Number of Quails	n	26	26	26	26
Period of the egg-laying records	Days	90	90	90	90
Age of the first egg laid	Days	55	40	45	45
Gross egg collection	1–30 days	178	435*	253	466*
	31–60 days	366	477*	236	484*
	61–90 days	409	576*	379	583*
	Total	953	1488*	867	1533*
Egg-laying intensity	Percentage	41.3	64.4*	40.0	69.0*

* indicates a statistically significant difference compared with the control group (p < 0.05). C.F. 2 = Commercial feed No. 2, CG = Control group, EG = Experimental group.

The age of first laying was earlier in the EG (40 days at ECO-KO; 45 days at JEBE) compared to the CG (55 days at ECO-KO; 45 days at JEBE) (p = 0.015). Egg-laying intensity was also significantly higher in the EG (64.4% at ECO-KO, 69.0% at JEBE) compared to CG (41.3% and 40.0%, respectively). Mortality during the laying phase was minimal (<5% per farm), indicating the safety of the phytobiotic feeds.

### Egg morphology

Morphological traits of eggs (Table S1) revealed that EG eggs were slightly heavier (12.75 g vs. 12.62 g) with increased albumen weight (6.40 g vs. 6.16 g) compared to CG. However, EG eggs exhibited reduced shell weight (1.84 g vs. 1.96 g), a lower shape index (73.28% vs. 75.39%), and lighter yolk pigmentation (4.17 vs. 4.67, p = 0.034). These results suggest that phytobiotic supplementation modifies certain egg quality parameters.

**Table S1:** Morphological characteristics of quail eggs (mean ± SEM) under different feeding regimes.

Indicator	ECO-KO		JEBE	
	
	CG (C.F. 2)	EG (starter + grower + layer)	CG (C.F. 2)	EG (starter + grower + layer)
Egg weight (g)	12.62 ± 0.42	12.75 ± 0.39	12.4 ± 0.65	13.23 ± 0.51
White weight (g)	6.16 ± 0.29	6.4 ± 0.27	6.55 ± 0.35	6.49 ± 0.65
Yolk weight (g)	4.50 ± 0.19	4.51 ± 0.18	4.29 ± 0.40	4.95 ± 0.34
Shell weight (g)	1.96 ± 0.04	1.84 ± 0.12	1.56 ± 0.11	1.8 ± 0.05
Relative weights (%)				
Protein	48.76 ± 1.66	50.17 ± 1.33	52.79 ± 0.49	48.74 ± 3.45
Yolk	35.72 ± 1.39	35.43 ± 1.21	34.4 ± 1.35	37.57 ± 2.97
Shell	15.57 ± 0.54	14.4 ± 0.77	12.81 ± 1.31	13.69 ± 0.75
Protein/yolk ratio	1.38 ± 0.10	1.43 ± 0.08	1.54 ± 0.06	1.35 ± 0.19
Egg diameter (mm)				
Big	3.52 ± 0.07	3.67 ± 0.09	3.43 ± 0.05	3.55 ± 0.08
Small	2.65 ± 0.05	2.68 ± 0.04	2.67 ± 0.07	2.63 ± 0.04
Egg shape index (%)	75.39 ± 1.18	73.28 ± 1.39	77.67 ± 1.10	74.26 ± 1.56*
Shell strength (μm)	1.55 ± 0.13	1.37 ± 0.15	1.59 ± 0.18	1.28 ± 0.11
Yolk color score	4.67 ± 0.26	4.17 ± 0.20*	3.5 ± 0.42	3.33 ± 0.52

* indicates a statistically significant difference compared with the control group (p < 0.05), C.F. 2 = Commercial feed no. 2, CG = Control group, EG = Experimental group, SEM = Standard error of the mean.

### Correlations between egg traits

Correlation analysis (Table S2) indicated strong positive associations between egg weight and protein weight in both CG and EG (r = 0.99 and 0.85, respectively). However, egg weight and yolk weight were strongly correlated in CG (0.97) but weakly in EG (0.24). Negative correlations were observed between egg weight and shell weight across both groups. In ECO-KO birds, yolk color correlated positively with yolk weight in CG (r = 0.74) but not in EG. These findings suggest that feeding regimes influence interrelationships among egg traits.

**Table S2:** Correlation coefficients between the morphological parameters of quail eggs at the JEBE and ECO-KO farms.

Indicator	JEBE		EKO-KO	
	
	CG	EG	CG	EG
Egg weight/protein weight (g)	0.99	0.84	0.85	0.80
Egg weight/yolk weight (g)	0.97	0.24	0.59	0.27
Egg weight/shell weight (g)	−0.81	−0.37	−0.30	−0.60
Diameter large/small (mm)	0.77	0.32	0.67	0.22
Yolk color/weight (score)/(g)	0.23	0.09	0.74	0.04

CG = Control group, EG = Experimental group, Bold values show the strong correlations (≥0.70).

### Chemical composition of eggs

The chemical profile (Table S3) showed minor differences between groups. CG eggs had slightly higher fat (11.83%) and protein content (13.04%) compared with EG eggs (11.28% fat and 12.54% protein). Conversely, EG eggs contained more carbohydrates (0.64% vs. 0.59%) and ash (1.47% vs. 1.26%). These results indicate subtle shifts in egg nutrient composition due to phytobiotic feeding.

**Table S3:** Chemical composition of quail eggs (mean ± SEM, % mass fraction) under different feeding regimes.

Indicator (%)	CG (JEBE + EKO-KO)	EG (JEBE + EKO-KO)
Fat mass fraction	11.83 ± 0.11	11.28 ± 0.12
Mass fraction of carbohydrates	0.59 ± 0.02	0.64 ± 0.19
Protein mass fraction	13.04 ± 0.74	12.54 ± 0.14
Mass fraction of the ash	1.26 ± 0.05	1.47 ± 0.04

CG = Control group, EG = Experimental group, SEM = Standard error of the mean.

### Mineral composition of eggs

Mineral analysis (Table S4) revealed minimal differences between CG and EG. Levels of potassium and phosphorus were nearly identical, whereas calcium was slightly higher in EG (52.34 mg/100 g) compared to CG (51.97 mg/100 g). Other minerals, including iron, copper, selenium, magnesium, and sodium, remained comparable. This suggests that phytobiotic-enriched feeds do not significantly alter egg mineral profiles.

**Table S4:** Mineral composition of quail eggs (mean ± SEM, mg/100 g) under different feeding regimes.

Parameter (mg/100 g)	CG (JEBE + EKO-KO)	EG (JEBE + EKO-KO)
Potassium	141 ± 0.15	141.4 ± 0.26
Phosphorus	199.6 ± 0.56	200.1 ± 0.87
Iron	3.01 ± 0.03	3.02 ± 0.04
Copper	0.09 ± 0.004	0.09 ± 0.004
Selenium	0.01 ± 0.002	0.01 ± 0.0007
Calcium	51.97 ± 0.38	52.34 ± 0.66
Magnesium	14.93 ± 0.06	14.31 ± 0.23
Sodium	157.34 ± 0.30	157.63 ± 0.55

CG = Control group, EG = Experimental group, SEM = Standard error of the mean.

### Summary of findings

**Figure 4:**
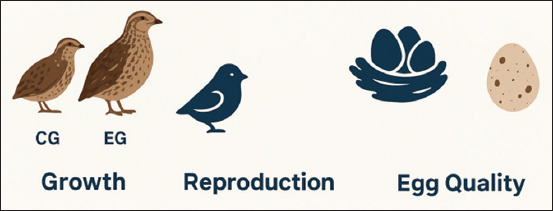
Key findings of the phytobiotic feeding trial in Manchurian quails

Overall, phytobiotic-enriched feeds enhanced survival, growth performance, feed efficiency, and egg productivity in Manchurian quails without adversely affecting health or egg mineral composition. Minor modifications were observed in egg morphology and nutrient composition. Figure 4 illustrates the main outcomes, showing superior growth, earlier onset of laying, improved laying intensity, and enhanced product quality in EG compared to CG.

## DISCUSSION

### Nutrient composition and mechanisms of action

The present study reveals that commercial and experimental quail feeds exhibit significant differences in nutrient composition, with Grower feed containing the highest crude protein (26.9%) and metabolic energy (3,371 kcal). This nutrient richness likely enhanced growth and feed efficiency through multiple mechanisms, including improved gut morphology, gut microbiota modulation, increased digestive enzyme secretion, and antioxidant activity, all of which collectively support nutrient use. Several related studies provide strong support for these findings. Higher levels of crude protein and metabolizable energy in quail diets directly improve growth rates and feed efficiency. For instance, studies by Balji *et al*. [9], Ashour *et al*. [33], and Dn and Andri [34] on laying quails demonstrate that diets enriched with phytobiotics and optimal protein-energy balance lead to better carcass yields and FCRs, aligning with the present findings. Compared with other quail trials using different phytobiotics, such as ginger at 0.5%–1.5% or frankincense oil at 200 mg/kg, the dosage levels in the present study were comparable, indicating consistent efficacy across plant-derived feed additives. The present study’s Grower feed, with even higher protein and energy, aligns with these optimal ranges and supports superior performance. Phytobiotics likely enhance productivity by improving gut morphology, nutrient absorption, antioxidant activity, reducing oxidative stress, and modulating gut microbiota and immunity.

### Growth performance

The present study demonstrates that quails fed phytobiotic Grower feed exhibited significantly superior growth compared with those fed commercial feed, achieving a 22% higher body weight gain and relative gains of 415.5% versus 297.5% (p = 0.002). Enhanced daily gains, notably 14.2 g at 28 days, underscore phytobiotics’ strong potential as growth promoters in quail farming systems. Multiple studies have confirmed that phytobiotic supplementation leads to significant improvements in quail live weight and daily gains. For example, one experiment found that adding phytobiotics increased live weight by 5.6%–14.0% depending on dosage and absolute live weight gains by 5.8%–14.5% compared to controls, with the highest dose group showing the most significant improvements [35]. These results are consistent with the present study’s observation of a 22% greater body weight gain and superior daily gains, underscoring the phytobiotics’ robust effect on growth. Mechanistically, these gains can be attributed to enhanced digestive enzyme activity and improved antioxidant status, thereby reducing metabolic stress and supporting protein accretion. Similar to our study, Karkach and Moiseenko [36] have also observed improvements in quail live weight indicators following the use of phytobiotic-enriched feeds. They determined that the trend of increased live weight in quails additionally fed ginger was 12.6%–17.1% higher in weeks 5 and 6 compared with the CG. Throughout the entire fattening period, the average daily weight gain in the groups receiving phytobiotic feeds exceeded that of the CG by 0.56 g (10.5%) and 0.69 g (12.9%), respectively [36].

### Egg production and reproductive performance

This study demonstrates that phytobiotic-enriched feeds significantly enhance egg production and laying intensity in quails. The EG produced markedly more eggs and laid earlier with higher intensities. These outcomes highlight phytobiotics’ ability to enhance reproductive performance and efficiency in commercial quail operations. The addition of phytobiotics, which are plant-derived feed additives with antimicrobial and antioxidant properties, has been shown to enhance egg production, mass, and quality. Chudak and Lebid [37] reported that phytobiotic supplementation increased total egg collection by up to 6.7%, improved egg mass and protein content, and boosted yolk and shell quality compared to controls. These improvements are consistent with the present study’s findings that phytobiotic-enriched feeds promote better productivity. Integrating alternative protein sources, such as fermented *Leucaena leucocephala* leaf meal, can also enhance nutrient intake, egg production, and egg quality in quails, especially when antinutritional factors are mitigated through fermentation. Optimal inclusion levels (4%–8%) increased energy and protein intake, improved egg quality, and reduced FCRs without increasing total feed intake [38]. This supports the idea that nutrient-rich, well-formulated feeds (including phytobiotics) maximize production efficiency. The magnitude of egg production improvements in this study was slightly higher than that in other trials with similar phytobiotic doses, likely due to the synergistic effects of the nutrient-rich Grower feed combined with phytobiotics. In terms of egg quality, phytobiotic-fed quails produced eggs with slightly altered morphology (heavier albumen, lighter shell, and reduced yolk pigmentation). These changes suggest modified nutrient allocation between egg components, possibly due to improved gut health and oviduct protein synthesis. Similar results were obtained, including the use of dried wormwood powder in quail compound feed at a concentration of 0.5%–1.5%, which had a positive effect on egg productivity indicators. The total egg yield increased by 2.6%–5.6% in the EG [39]. In addition, the egg mass increased by 5.6%–12.9%, whereas the feed costs decreased by 2.8%. In terms of egg quality parameters, an increase was observed in the absolute mass of the yolk (by 3.0%–8.3%), egg white (by 2.6%–6.5%), and shell (by 0.6%–1.2%), whereas the egg index decreased by 0.9%–4.1% [40].

### Egg morphology and quality

The present study shows that phytobiotic-fed quails laid slightly heavier eggs with higher albumen weight but reduced shell weight and lower yolk color score. A lower shape index suggests subtle changes in the geometry of eggs. These findings suggest that phytobiotics may regulate nutrient allocation between egg components by improving gut health and enhancing protein synthesis in the oviduct. Beyond growth, phytobiotics improve egg production and quality. Supplemented quails exhibited increased egg mass, protein, and yolk content, improved shell quality, higher laying rates, and higher gross egg collection [41]. Although the present study focuses on growth, these findings suggest that phytobiotic feed additives can provide comprehensive benefits to quail productivity. Research in broilers and quails indicates that phytobiotics can match or even surpass the growth-promoting effects of traditional antibiotics without the associated risks of antibiotic resistance. In some studies, combinations of phytobiotics and probiotics yielded the best results; however, phytobiotics alone consistently outperformed controls and antibiotic-only groups in terms of live weight gain and feed conversion [42]. In addition, the improvements in egg weight and albumen observed in this study may be related to phytobiotic-induced enhanced feed conversion and metabolic efficiency, as reported in other poultry studies. Antioxidant and antimicrobial properties reduce oxidative stress and improve gut health, facilitating better nutrient absorption, which is critical for egg formation [43]. In summary, the increase in egg and albumen weight observed with phytobiotic supplementation in this quail study is consistent with broader evidence that phytobiotics enhance egg production in laying birds by improving digestive health, antioxidant status, and protein synthesis mechanisms.

### Correlations among egg traits

The present study revealed strong positive correlations between egg weight and protein weight, whereas phytobiotic-fed groups demonstrated weak correlations for yolk color, suggesting altered pigment deposition. The negative relationships between egg and shell weights imply resource trade-offs. Thus, phytobiotic feeding subtly modifies the internal interrelations among egg morphological traits. Additional literature reviews and experimental reports indicate that phytobiotics, such as thyme, garlic, and ginger derivatives, generally enhance egg production, egg size, shell thickness, and egg mass in laying birds by improving mineral absorption (particularly phosphorus and calcium) and modulating gut health and metabolism. However, the effects on egg shape and yolk color can vary depending on the specific plant compounds used, their concentrations, and bird species [44]. Specifically, phytobiotics contain bioactive ingredients such as phenolic acids, gingerols, and tea polyphenols that exhibit antimicrobial and antioxidant effects, which can enhance the integrity of the ovarian and uterine oviduct and improve digestive efficiency. This leads to enhanced protein synthesis and albumen secretion, thereby increasing albumen weight and improving egg quality parameters such as Haugh unit (a measure of albumen quality). For example, green tea polyphenols upregulate proteins related to albumen quality, increasing ovalbumin content and reducing the degradation of thick albumen proteins, which supports higher albumen weight and better egg quality [45]. In addition, a study demonstrated that including ginger root extract in compound feed did not negatively affect growth parameters. Still, it strengthened the immune system, suppressed *Escherichia coli* growth, and promoted beneficial bacterial growth. Their findings also indicated that feeding laying quails with 500 mg/kg of ginger powder or 200 mg/kg of frankincense oil as a phytobiotic improved reproductive performance and overall productivity compared with the CG [31].

### Chemical composition of eggs

The present study reveals that eggs from phytobiotic-fed quails contained slightly lower fat and protein contents, with modest increases in carbohydrate and ash contents. These minor shifts suggest that phytobiotic supplementation can influence the chemical composition of eggs, possibly by improving mineral absorption and slightly modulating lipid and protein deposition. First, the observed modest reduction in egg fat and protein content corresponds with reports that phytobiotics can modulate egg chemical composition by influencing nutrient metabolism and absorption. For example, a study by Kachanov and Poberezhets [46] investigated phytobiotic additives in quail feed found increases in egg protein and yolk weight, as well as improvements in egg quality parameters such as yolk height and shell weight, indicating enhanced mineral content and egg structure integrity. Another study by Ali [47] revealed that the slight reduction in fat and protein content in phytobiotic-fed quail eggs somewhat contrasts with the general finding that quail eggs naturally have higher protein and fat contents than chicken eggs. However, the reduction is minor and could reflect a shift in nutrient partitioning due to phytobiotic effects on metabolism or gut microbiota. Study by Rabelo-Ruiz *et al*. [48] with allium-based phytobiotics (garlic and onion compounds) in laying hens demonstrated increased egg production and size, linked to beneficial shifts in gut microbiota that improve nutrient digestibility. This microbial modulation may partly explain changes in egg composition by altering nutrient absorption and metabolism. While the present study noted reduced fat content, other research on quail eggs showed a complex fatty acid profile rich in beneficial unsaturated fatty acids [49]. Phytobiotics can possess prebiotic properties, stimulating the growth of beneficial bacteria that produce enzymes, including those that aid in the absorption of minerals, thereby enhancing digestion and nutrient assimilation [50]. In summary, phytobiotics enhance mineral absorption in quails by promoting a healthier intestinal environment, improving gut morphology, and facilitating better nutrient uptake and retention, resulting in increased mineral content (ash) in eggs and improved egg quality. The study by Wengerska *et al*. [51] demonstrated that adding 10% fermented rapeseed meal had the most favorable impact on qualitative egg characteristics, including egg mass, specific gravity, yolk index, yolk color, and egg white pH. These findings align with the results reported in our research.

### Mineral composition of eggs

The present study demonstrates that the mineral composition of quail eggs remains essentially unchanged, particularly with phytobiotic feeding, with nearly identical potassium and phosphorus levels between groups. A slightly higher calcium level in experimental eggs suggests that phytobiotics do not disrupt mineral profiles. Overall, phytobiotic productivity gains come without compromising egg mineral nutrition. Similarly, research combining probiotics and acidifiers in late-laying quails showed improvements in egg mass and nutrient value but found no significant differences in ash content (a proxy for mineral content) across treatments, indicating that the mineral composition remains stable despite dietary supplementation [52]. Phytobiotics are known to improve gut health and nutrient absorption, which can enhance mineral bioavailability; however, this does not necessarily translate into altered mineral concentrations in eggs, as egg mineral homeostasis is tightly regulated to maintain egg quality [53]. In contrast, some studies suggest that phytobiotics can enhance mineral absorption and accumulation in eggshells and tissues, potentially improving calcium and phosphorus levels in these areas. However, these effects may be more pronounced in eggshell quality than in the internal mineral content of the egg [54]. Gut health improvements from phytobiotics can positively influence mineral deposition in quail eggs through several mechanisms. Enhanced nutrient absorption: Phytobiotics improve gut morphology by increasing villus height and the villus height to crypt depth ratio, thereby expanding the intestinal surface area available for nutrient absorption, including minerals such as calcium and phosphorus, which are critical for egg formation [55]. This improved absorptive capacity facilitates more efficient uptake of minerals from the diet. Furthermore, the antioxidant and antimicrobial effects of phytobiotics protect the intestinal epithelium, enhancing mineral retention and bioavailability without altering systemic mineral homeostasis.

### Overall significance and novelty

Overall, this study contributes to the growing evidence that phytobiotics enhance quail growth, reproduction, and egg quality primarily through gut health modulation, antioxidant protection, and improved nutrient use. Importantly, these benefits occur without compromising mineral composition, supporting phytobiotics as safe and sustainable alternatives to antibiotics in modern quail farming.

The novelty of this work lies in its focus on Manchurian quails, a breed less frequently studied than Japanese or Texas quails, and in its evaluation of phytobiotics within a multi-phase feeding regimen. By demonstrating that phytobiotic-enriched Grower feed optimizes both growth and reproductive performance in this specific breed, this study advances the current understanding of breed-specific responses and provides practical guidance for sustainable quail farming practices.

### Limitations

A key limitation of this study is the absence of biochemical analyses of muscle tissue, including protein and lipid profiles, which would have provided a more comprehensive assessment of meat quality. As outlined in the methodology, bird slaughter was not permitted at the experimental farms due to ethical restrictions. Additional limitations include the lack of long-term reproductive assessments beyond the 90-day trial, the relatively small number of farms involved, which may limit the generalizability of findings, and the absence of an economic feasibility or cost-benefit evaluation of phytobiotic-enriched feeds. Nevertheless, no adverse events were recorded, and mortality remained within physiological norms, confirming the safety of the phytobiotic feeding regimen.

## CONCLUSION

This study demonstrated that phytobiotic-enriched multiphase feeds significantly improved both growth and reproductive performance in Manchurian quails. Birds fed experimental diets achieved a 22% higher body weight gain (186.3 g vs. 135.3 g) and markedly better feed efficiency (FCR: 2.05 vs. 2.45, p = 0.001). Egg production also improved substantially, with earlier onset of laying, higher egg counts (1533 vs. 867 at JEBE; 1488 vs. 953 at ECO-KO), and greater laying intensity (64.4%–69.0% vs. 40.0%–41.3%). While egg morphology showed slight modifications, including increased albumen, lighter yolk pigmentation, and reduced shell weight, the chemical and mineral composition remained largely stable, and mortality stayed below 5%, confirming the safety of phytobiotic supplementation.

These findings confirm the potential of phytobiotic-enriched feeds as a sustainable alternative to antibiotic-based growth promoters in quail farming. By enhancing growth, feed utilization, and reproductive performance without compromising egg mineral quality, phytobiotics can improve farm profitability and meet consumer demand for safe, antibiotic-free poultry products. Their integration into commercial production systems may reduce antimicrobial resistance risks and promote long-term sustainability in animal agriculture.

The strengths of this study include the use of a large sample size (1600 birds) across two independent commercial farms, the evaluation of a multiphase feeding regimen tailored to distinct growth and reproductive phases, and a comprehensive assessment of growth, feed efficiency, egg productivity, morphology, and composition. Laboratory analyses were conducted using standardized AOAC, ISO, and GOST protocols, ensuring methodological rigor.

Future research should expand the scope of phytobiotic evaluation by conducting biochemical analyses of muscle tissue to assess meat quality, assessing long-term reproductive outcomes beyond 90 days and across multiple production cycles, performing economic cost-benefit analyses to evaluate commercial viability, and investigating molecular mechanisms such as gut microbiome shifts, immune modulation, and oxidative stress markers. Extending studies to other quail breeds and poultry species will further enhance generalizability.

In conclusion, phytobiotic-enriched multiphase feeding offers a safe, effective, and sustainable strategy to enhance growth efficiency, feed utilization, and reproductive output in Manchurian quails. By demonstrating improvements in both performance and product quality without adverse effects, this study advances the evidence base for phytobiotics as natural alternatives to antibiotics. The novelty of applying phytobiotics in a multiphase feeding system for Manchurian quails provides valuable insights for breed-specific nutrition and contributes to the development of eco-friendly and profitable quail farming practices.

## DATA AVAILABILITY

All data generated or analyzed during this study are included in this published article**.**

## AUTHORS’ CONTRIBUTIONS

DZ, BA, GA, DS, BK, SR, and AZ: Study conception and design, data collection and analysis, interpretation of results, and drafted and edited the manuscript. All authors have read and approved the final version of the manuscript.

## References

[1] Fathi MM, Galal A, Al-Homidan I, Abou-Emera O.K, Rayan G.N (2021). Residual feed intake:A limiting economic factor for selection in poultry breeding programs. Ann. Agric. Sci.

[2] Indrayani I, Nurhayati N, Rahman N.A (2024). Economic analysis of laying quail farming business in Barangin district, Sawahlunto city. IOP. Conf. Ser. Earth Environ. Sci.

[3] Bocharova P.A, Bachinskaya V.M, Bachinskaya N.A (2023). The effect of feed additives on the amino acid composition of quail eggs. Probl. Vet. Sanit. Hyg. Ecol.

[4] Shalome G.O, Nojuvwevwo L.I (2021). Quail husbandry and welfare systems at Songhai-Delta farm:Profitability of enterprise. Niger. J. Anim. Prod.

[5] Semyonov S, Safonov V, Vencova I, Proskurina I (2024). Using additives to improve the effectiveness of rations in quail farming for meat. BIO Web. Conf.

[6] Kinyua M (2022). Factors influencing quail farming:A critical literature review. Anim. Health J.

[7] El-Saadony M.T, Abd El-Hack M.E, Swelum A.A, Al-Sultan S.I, El-Ghareeb W.R, Hussein E.O.S, Ba-Awadh H.A, Akl B.A, Nader MM (2021). Enhancing quality and safety of raw buffalo meat using the bioactive peptides of pea and red kidney bean under refrigeration conditions. Ital J. Anim. Sci.

[8] Zaikina A.S, Buryakov N.P, Buryakova M.A, Zagarin A.Y, Razhev A.A, Aleshin D.E (2022). Impact of supplementing phytobiotics as a substitute for antibiotics in broiler chicken feed on growth performance, nutrient digestibility, and biochemical parameters. Vet. Sci.

[9] Balji Y, Zhanabayeva D, Sultanayeva L, Yeszhanova G, Mussagiyeva D (2024). Effect of feed with extruded components and phytobiotics on quail. Sci. Horiz.

[10] Elzaher H.A.A, Ibrahim Z.A, Ahmed S.A, Salah A.S, Osman A, Swelum A.A, Suliman G.M, Tellez-Isaias G, Alagawany M, Abd El-Hack M.E (2023). Growth, carcass criteria, and blood biochemical parameters of growing quails fed *Arthrospira platensis* as a feed additive. Poult. Sci.

[11] Alagawany M, El-Hindawy M.M, Mohamed L.A, Bilal R.M, Soomro J (2022). The use of cold pressed oils as eco-friendly alternatives for antibiotics in high and low-CP diets of laying Japanese quail. Anim. Biotechnol.

[12] Obianwuna U.E, Chang X, Oleforuh-Okoleh V.U, Onu P.N, Zhang H, Qiu K, Wu S (2024). Phytobiotics in poultry:Revolutionizing broiler chicken nutrition with plant-derived gut health enhancers. J. Anim. Sci. Biotechnol.

[13] Deminicis R.G.D.S, Meneghetti C, De Oliveira E.B, Júnior A.A.P.G, Filho R.V.F, Deminicis B.B (2021). Systematic review of the use of phytobiotics in broiler nutrition. Rev. Ciênc. Agrovet.

[14] Kanbur G, Göçmen R, Cufadar Y (2023). Effect of dietary banana leaves (*Musa acuminata*) with multienzyme complex supplementation on intestinal content, serum biochemicals, egg production, and egg quality in laying quail. Livest. Sci.

[15] Lee S.A, Lopez D.A, Stein H.H (2022). Invited review -mineral composition and phosphorus digestibility in feed phosphates fed to pigs and poultry. Anim. Biosci.

[16] Reda F.M, Alagawany M, Mahmoud H.K, Aldawood N, Alkahtani A.M, Alhasaniah A.H, Mahmoud M.A, El-Saadony M.T, El-Kassas S (2024). Application of Naringenin as a natural feed additive for improving quail performance and health. J. Appl. Poult. Res.

[17] Kour G, Khan N, Sharma R.K, Mahajan V, Khandi S.A, Aït-Kaddour A, Bekhit A.A, Bhat Z.F (2025). Effect of phytogenic feed additives on egg quality and production potentialities of quails. Emerg. Anim. Species.

[18] Alagawany M, Abd El-Hack M.E, Farag M.R, Elnesr S.S, El-Kholy M.S, Saadeldin I.M, Swelum A.A (2018). Dietary supplementation of *Yucca schidigera* extract enhances productive and reproductive performances, blood profile, immune function, and antioxidant status in laying Japanese quails exposed to lead in the diet. Poult. Sci.

[19] Shin S, Chen S, Xie K, Duhun S.A, Ortiz-Cerda T (2025). Evaluating the anti-inflammatory and antioxidant efficacy of complementary and alternative medicines (CAM) used for management of inflammatory bowel disease:A comprehensive review. Redox. Rep.

[20] Singh J, Gaikwad D.S, Singh J, Yadav A (2020). Phytogenic feed additives in animal nutrition. Natural Bioactive Products in Sustainable Agriculture.

[21] Sultanayeva L, Karkehabadi S, Zamaratskaia G, Balji Y (2023). Tannins and flavonoids as feed additives in the diet of ruminants to improve performance and quality of the derived products. A review. Bulg. J. Agric. Sci.

[22] Rahman M.M, Sukor S.A, Umami N, Sukri S.A.M (2025). Effects of olive oil-treated diet and key lime juice-treated drinking water on intake and growth performance of quail. Vet. Integr. Sci.

[23] Lukanov H, Pavlova I, Genchev A (2023). Effect of fattening period duration on meat productivity of domestic quails from different productive types. Poult. Sci.

[24] Abd El-Ghany W.A (2020). Phytobiotics in poultry industry as growth promoters, antimicrobials and immunomodulators - a review. J. Worlds Poult. Res.

[25] El-Shall N.A, Awad A.M, El-Hack M.E.A, Naiel M.A.E, Othman S.I, Allam A.A, Sedeik M.E (2019). The simultaneous administration of a probiotic or prebiotic with live *Salmonella* vaccine improves growth performance and reduces fecal shedding of the bacterium in *Salmonella*-challenged broilers. Animals (Basel).

[26] Shahbakht R.M, Nomi Z.A, Kabeer S.W, Anwar M.Z, Shahid S, Imtiaz B, Younus G, Muneer M.H, Usman M, Rajput S.A, Abbas R.Z, Akhtar T, Asrar R, Khan A.M.A, Saeed Z (2024). The use of phytogenic feed additives in animal nutrition in the Pakistan scenario. Complementary and Alternative Medicine:Feed Additives.

[27] Gnanaraj P.T, Ezhil Valavan S, Bharathi A.A (2023). Effect of phytogenic feed additives on growth performance of Japanese quail. Biol. Forum. Int. J.

[28] Abou-Elkhair R, Selim S, Hussein E (2018). Effect of supplementing layer hen diet with phytogenic feed additives on laying performance, egg quality, egg lipid peroxidation and blood biochemical constituents. Anim. Nutr.

[29] Ibrayev B. K, Zhanabayev B. K, Bushmanov P. G &others. (n.d.). Patent No. KZ21292-A4.

[30] Vargas-Sánchez R.D, Ibarra-Arias F.J, Torres-Martínez B.D.M, Sánchez-Escalante A, Torrescano-Urrutia G.R (2019). Use of natural ingredients in the Japanese quail diet and their effect on carcass and meat quality. Review. Asian Australas J. Anim. Sci.

[31] Dosu G, Obanla T.O, Zhang S, Sang S, Adetunji A.O, Fahrenholz A.C, Ferket P.R, Nagabhushanam K, Fasina Y.O (2023). Supplementation of ginger root extract into broiler chicken diet:Effects on growth performance and immunocompetence. Poult. Sci.

[32] Council N.R (1994). Nutrition S on P. Nutrient Requirements of Poultry:1994. National Academies Press.

[33] Ashour E.A, Alabdali A.Y, Aldhalmi A.K, Taha A.E, Swelum A.A, Abd El-Hack M.E (2022). Impacts of varying dietary energy and crude protein levels on growth, carcase traits and digestibility coefficients of growing Japanese quail (*Coturnix Coturnix Japonica*) during the summer season. Ital. J. Anim. Sci.

[34] Dn E, Andri F (2023). Effect of dietary with different energy and protein levels on laying quails'performance. J. Ilmu Ternak Vet.

[35] Chudak R.A, Lebid Y.G (2024). Efficiency of feed utilization and growth of repair young quails with the use of a phytobiotic supplement. Sci. Messin. LNU Vet. Med. Biotech. Ser. Agric. Sci.

[36] Karkach P, Moiseenko K (2024). Productivity and meat quality of quail with the addition of garlic and ginger to the diet. Tehnol. Virobn. Pererobki Prod. Tvarinn.

[37] Chudak RA, Lebid Y.G (2025). Effect of phytobiotics on quail egg productivity. Sci. Messin. LNU Vet. Med. Biotech. Ser. Agric. Sci.

[38] Utami M.M.D, Akbar A (2025). Enhancing nutrient intake egg production and egg quality by fermented *Leucaena leucocephala* leaf meal in a diet of laying quail. Vet. World.

[39] Sychov M, Umanets D, Balanchuk I, Umanets R, Ilchuk I, Holubieva T (2024). Effect of feeding *Artemisia capillaris* on egg production and egg quality in quail. J. Anim. Sci. Technol.

[40] Cullere M, Singh Y, Pellattiero E, Berzuini S, Galasso I, Clemente C, Zotte A.D (2023). Effect of the dietary inclusion of *Camelina sativa* cake into quail diet on live performance, carcass traits, and meat quality. Poult. Sci.

[41] Naderi E, Akbari S.M, Manochehri H (2021). The effect of different levels of Lavender essential oil and bacitracin methylene disalicylate supplementation on performance, carcass traits, some blood parameters, small intestinal morphology and microflora in Japanese quails. Rev. Bras. Higiene Sanidade Anim.

[42] Ferdous M.F, Arefin M.S, Rahman M.M, Ripon M.M.R, Rashid M.H, Sultana M.R, Hossain M.T, Ahammad M.U, Rafiq K (2019). Beneficial effects of probiotic and phytobiotic as growth promoter alternative to antibiotic for safe broiler production. J. Adv. Vet. Anim. Res.

[43] Sharma M.K, Dinh T, Adhikari P.A (2020). Production performance, egg quality, and small intestine histomorphology of the laying hens supplemented with phytogenic feed additive. J. Appl. Poul. Res.

[44] Zamir S, Yousuf R, Ahmad T, Aziz Ur Rahman M, Mukhtar N, Ashraf M, Khalid M.F, Zafar M.A, Rehman ZU (2024). Impact of Phytobiotics on Poultry Health and Diseases.

[45] Obianwuna U.E, Oleforuh-Okoleh V.U, Wang J, Zhang H.J, Qi G.H, Qiu K, Wu S.G (2022). Natural products of plants and animal origin improve albumen quality of chicken eggs. Front. Nutr.

[46] Kachanov I.O, Poberezhets J.M (2025). Egg productivity of quails with the use of a probiotic supplement. Sci. Messin. LNU Vet. Med. Biotech. Ser. Agric. Sci.

[47] Ali MA (2019). A comparative study on nutritional value of quail and chicken eggs مجلة البحوث فی مجالات التربیة النوعیة,.

[48] Rabelo-Ruiz M, Ariza-Romero J.J, Zurita-González M.J, Martín-Platero A.M, Baños A, Maqueda M, Valdivia E, Martínez-Bueno M, Peralta-Sánchez JM (2021). *Allium*-based phytobiotic enhances egg production in laying hens through microbial composition changes in ileum and cecum. Animals.

[49] Tokuşoĝlu Ö (2006). The quality properties and saturated and unsaturated fatty acid profiles of quail egg:The alterations of fatty acids with process effects. Int. J. Food Sci. Nutr.

[50] Smolentsev S, Strelnikova I, Kislistina N, Gracheva O, Gasanov A, Amirov D, Mukhutdinova D.M, Tamimdarov B.F, Gugkaeva M.S, Tsugkieva Z.R, Ktsoeva I, Gertman A.M, Samsonova T.S (2020). Effectiveness of the use of biologically active substances in quail farming. J. Archaeol. Egypt Egyptol.

[51] Wengerska K, Czech A, Knaga S, Drabik K, Próchniak T, Bagrowski R, Gryta A, Batkowska J (2022). The quality of eggs derived from Japanese quail fed with the fermented and non-fermented rapeseed meal. Foods.

[52] Lokapirnasari W.P, Al-Arif M.A, Hidayatik N, Safiranisa A, Arumdani D.F, Zahirah A.I, Yulianto A.B, Lamid M, Marbun T.D, Lisnanti E.F, Baihaqi Z.A, Khairullah A.R, Kurniawan S.C, Pelawi E.B.S, Hasib A (2024). Effect of probiotics and acidifiers on feed intake, egg mass, production performance, and egg yolk chemical composition in late-laying quails. Vet. World.

[53] Alagawany M, Elnesr S.S, Farag M.R, Abd El-Hack M.E, Barkat R.A, Gabr A.A, Foda M.A, Noreldin A.E, Khafaga A.F, El-Sabrout K, Elwan H.A.M, Tiwari R, Yatoo M.I, Michalak I, Di Cerbo A, Dhama K (2021). Potential role of important nutraceuticals in poultry performance and health - a comprehensive review. Res. Vet. Sci.

[54] Salazar I, Rodríguez R, Aroche R, Valdivié M, Martínez Y (2021). Phytobiotic effect of *Jatropha curcas* leaf powder on productivity, egg quality and blood biochemistry of laying quails. Cuban J. Agric. Sci.

[55] Marwi F, Sjofjan O, Mutaqin A, Natsir M.H (2021). The effect of Phytobiotics supplementation and magnetized drinking water on production performance and egg quality of laying hens. J. Iilu Teknol. Hasil Ternak.

